# Lung-thorax compliance measured during a spontaneous breathing trial is a good index of extubation failure in the surgical intensive care unit: a retrospective cohort study

**DOI:** 10.1186/s40560-018-0313-9

**Published:** 2018-07-31

**Authors:** Yugo Okabe, Takehiko Asaga, Sayuri Bekku, Hiromi Suzuki, Kanae Kanda, Takeshi Yoda, Tomohiro Hirao, Gotaro Shirakami

**Affiliations:** 1 Department of Anesthesiology, Faculty of Medicine, Kagawa University, 1750-1 Ikenobe, Miki-Cho, Kagawa 761-0793 Japan; 2 Department of Hygiene, Faculty of Medicine, Kagawa University, Miki-Cho, Kagawa Japan; 3 Department of Public Health, Faculty of Medicine, Kagawa University, 1750-1 Ikenobe, Miki-Cho, Kagawa 761-0793 Japan; 4 Department of Health and Sports Science, Kawasaki University of Medical Welfare, 228 Matushima, Kurasiki-shi, Okayama, 701-0193 Japan

**Keywords:** Lung and thorax compliance, Spontaneous breathing trial, Extubation failure, Proportional assist ventilation

## Abstract

**Background:**

Extubation failure is associated with mortality and morbidity in the intensive care unit. Ventilator weaning protocols have been introduced, and extubation is conducted based on the results of a spontaneous breathing trial. Room for improvement still exists in extubation management, and additional objective indices may improve the safety of the weaning and extubation process. Static lung-thorax compliance reflects lung expansion difficulty that is caused by several conditions, such as atelectasis, fibrosis, and pleural effusion. Nevertheless, it is not used commonly in the weaning and extubation process. In this study, we investigated whether lung-thorax compliance is a good index of extubation failure in adults even when patients pass a spontaneous breathing trial.

**Methods:**

In a single-center, retrospective cohort study, patients over 18 years of age were mechanically ventilated, weaned with proportional assist ventilation, and underwent a spontaneous breathing trial process in surgical intensive care units of Kagawa University Hospital from July 2014 to June 2016. Extubation failure was the outcome measure of the study. We defined extubation failures as when patients were reintubated or underwent non-invasive positive-pressure ventilation within 24 h after extubation. Receiver operating characteristic (ROC) curve analysis was performed to evaluate the clinical involvement of several parameters. The area under the curve (AUC) was calculated to assess the discriminative power of the parameters.

**Results:**

We analyzed 173 patients and compared the success and failure groups. Most patients (162, 93.6%) were extubated successfully, and extubation failed in 11 patients (6.4%). The averages of lung-thorax compliance values in the success and failure groups were 71.9 ± 23.0 and 43.3 ± 14.6 mL/cmH_2_O, respectively, and were significantly different (*p* < 0.0001). In the ROC curve analysis, the AUC was highest for lung-thorax compliance (0.862), followed by the respiratory rate (0.821), rapid shallow breathing index (0.781), Acute Physiology and Chronic Health Evaluation II score (0.72), heart rate (0.715), and tidal volume (0.695).

**Conclusions:**

Lung-thorax compliance measured during a spontaneous breathing trial is a potential indicator of extubation failure in postoperative patients.

## Background

Extubation failure is associated with mortality and morbidity in the intensive care unit (ICU) [1–6]. Ventilator weaning and extubation were previously based on the experience of the intensivist, but in recent years, ventilator weaning protocols have been introduced, and extubation is conducted based on the results of a spontaneous breathing trial (SBT) [7–9]. SBT is a test to determine whether a patient can tolerate the condition without the support of mechanical ventilation. Close observation and objective judgment contribute to shortening mechanical ventilation duration and reducing the reintubation rate [7–9]; however, the reintubation rate is still 11–19% [1–4, 10–15]. Room for improvement still exists in extubation management, and additional objective indices may increase the safety of the weaning and extubation process.

Static lung-thorax compliance (LTC), which is calculated by the formula: tidal volume (mL)/(pressure measured from the onset of end-inspiratory occlusion − positive end-expiratory pressure) (cmH_2_O) [16–18], is a candidate for an index that can help to more safely extubate patients. LTC reflects the difficulty of lung expansion that is caused by several conditions such as atelectasis, fibrosis, pleural effusion, intrapulmonary fluid retention, or a decrease in compliance due to obesity [17–19]. Monitoring LTC is useful because intensivists can evaluate the conditions of the lung and respiratory muscles [20, 21]. However, LTC is not commonly used in the weaning and extubation process because the measurement of LTC under spontaneous breathing is possible only under proportional assist ventilation (PAV) [16]. Thus, the relationship between LTC during spontaneous breathing and extubation failure is not clear.

PAV is a mode that assists ventilation in proportion to the instantaneous effort of the patient’s breathing [22]. In this mode, LTC can be measured with less stress for the patients [16]. Because PAV is superior to pressure support ventilation (PSV), which is synchronized with spontaneous breathing [23–25], intensivists can reduce sedative use [26] and judge an SBT more precisely [27–29]. In this study, we investigated whether LTC is a good index of extubation failure among adults who passed the SBT.

## Methods

### Study population

**Fig. 1:**
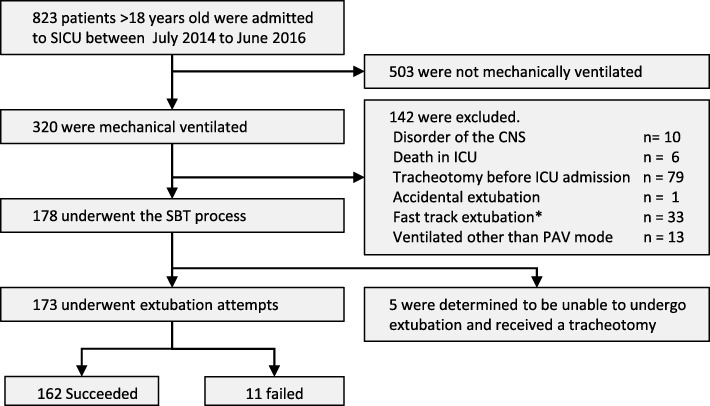
Number of patients included and excluded from the study. A total of 823 patients older than 18 years of age were admitted to the SICU. Among them, 503 patients were not mechanically ventilated during the SICU stay. According to the exclusion criteria, 142 patients were excluded. A total of 178 patients underwent the SBT process; however, 5 patients underwent a tracheotomy. Finally, we analyzed 173 patients and compared the success and failure groups. Most patients (162, 93.6%) were extubated successfully, and 11 (6.4%) failed the extubation. *Fast track extubation: extubation without SBT for patients with good postoperative condition; SICU: surgical intensive care unit; CNS: central nervous system; ICU: intensive care unit; PAV: proportional assist ventilation; SBT: spontaneous breathing trial

We conducted a single-center, retrospective cohort study involving patients over 18 years of age who were admitted to the surgical intensive care unit (SICU) in Kagawa University Hospital from July 2014 to June 2016. Patients who were ventilated mechanically, weaned with PAV, and underwent a SBT process were included in the analysis. The following patients were excluded because the SBT process was not conducted: patients who had disorders in their central nervous system, patients who died before extubation, patients who underwent a tracheotomy before SICU admission, patients who were extubated accidentally, and patients with a good postoperative condition who were extubated without SBT (Fast track extubation). In addition, patients who were subjected to ventilation modes other than PAV for staff education were also excluded (Fig. 1).

### SBT process

**Fig. 2:**
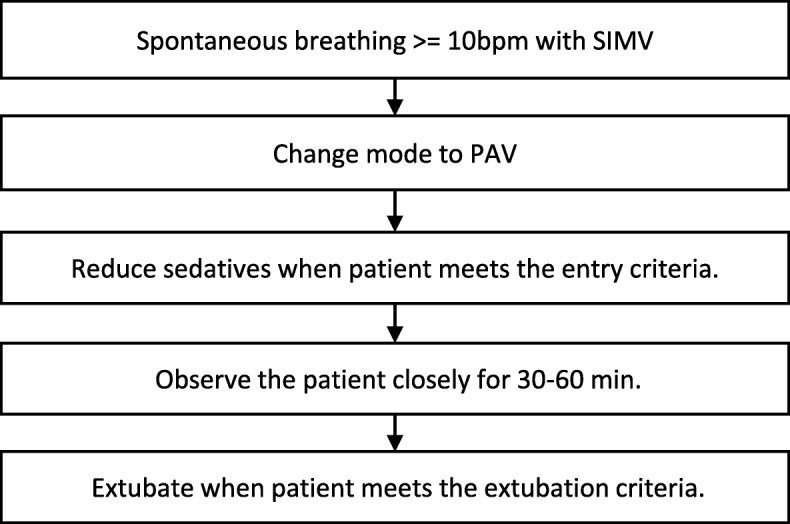
Flowchart of the SBT process. Intensivists assessed the spontaneous breathing ability of the patient before the SBT process. When the patient exhibited spontaneous breathing of 10 times per minute or more, PAV was initiated. Under close observation and when the patient met the entry criteria, sedative drugs were reduced until the RASS was − 2 to 0. Intensivists observed the patient for 30–60 min and determined whether to extubate when the patient met the extubation criteria. SIMV: synchronized intermittent mandatory ventilation; PAV: proportional assist ventilation; SBT: spontaneous breathing trial

Intensivists assessed the spontaneous breathing ability of the patient before starting the SBT process (Fig. 2). The ventilation mode was changed to synchronized intermittent mandatory ventilation (SIMV) at the time of ICU admission. We used propofol and dexmedetomidine hydrochloride for sedation and maintained the Richmond Agitation-Sedation Scale (RASS) between − 3 and − 1 [30]. We also used a continuous infusion of fentanyl 10–100 mcg/h, peripheral nerve block (PNB), epidural anesthesia, and nonsteroidal anti-inflammatory drugs (NSAIDs) for analgesia. When the patient exhibited spontaneous breathing of ten times per minute or more, PAV was initiated. We adjusted the support rate (15–30%) so that the patient’s work of breathing (WOB) was maintained in a comfortable range (0.3–0.7 J/L). Under close observation and when the patient met the entry criteria (Table 1), sedative drugs were reduced until the RASS was − 2 to 0. Intensivists observed the patient for 30–60 min [31] and determined whether to extubate when the patient met the extubation criteria (Table 1) [32]. If patients did not meet the criteria, PAV was continued, and ICU members, including intensivists, attending physicians, anesthesiologists, and ICU nurses, discussed whether to wait until the patient’s status improved or a tracheotomy was performed [33]. The PB 840 ventilator (Covidien, USA) was employed to apply the PAV mode for patients. After extubation, all patients were given a high-flow nasal cannula (HFNC) or oxygen mask. If the patient failed to maintain with HFNC, we decided whether to use non-invasive positive-pressure ventilation (NPPV) or reintubate.

**Table 1:** Criteria for the SBT process

Entry criteria	
SpO_2_	≥ 94% with FiO_2_ ≤ 0.5, PEEP ≤ 7 cmH_2_O, support of WOB ≤ 40%
PaO_2_	≥ 70 mmHg
Respiratory acidosis	No acidosis
Heart rate	≤ 120 bpm
Dopamine	≤ 5 mcg/kg/min
Dobutamine	≤ 5 mcg/kg/min
Noradrenaline	≤ 0.05 mcg/kg/min
Hemoglobin	≥ 8 g/dl
Electrolyte abnormality	No abnormality
Extubation criteria	
Consciousness	
Richmond Agitation-Sedation Scale	− 2 to 0
Confusion assessment method for the ICU	Negative
Respiration	
Respiratory rate	≤ 30/min
RSBI	< 105
Labored breathing	No
Increased WOB	No
Gas exchange	
SpO_2_	≥ 94%
PaO_2_	≥ 70 mmHg
pH	≥ 7.32
PaCO_2_	≤ 45 mmHg
Circulation	
Heart rate	≤ 120/min
Systolic blood pressure	80 to 180 mmHg

*SBT* spontaneous breathing trial, *FiO*_*2*_ inspired oxygen fraction, *PEEP* positive end-expiratory pressure, *WOB* work of breathing, *SpO*_*2*_ arterial oxygen saturation, *PaO*_*2*_ partial pressure of arterial oxygen, *RSBI* rapid shallow breathing index, *PaCO*_*2*_ partial pressure of arterial carbon dioxide

### Outcome and parameters

Extubation failure was the outcome measure of the study. We defined extubation failure as when patients were reintubated or when NPPV was conducted within 24 h after extubation. As potential factors influencing the outcome, sex, age, body mass index (BMI) at admission, type of surgery, emergency surgery, ventilation period, number of SBT, use of HFNC and NPPV, and Acute Physiology and Chronic Health Evaluation II (APACHE II) scores [34] at SICU entrance were collected through the electronic medical record. Parameters that can be monitored during the weaning process, such as the heart rate (HR), respiratory rate (RR), tidal volume (TV), rapid shallow breathing index (RSBI), positive end-expiratory pressure (PEEP), arterial oxygen saturation (SpO_2_), end-tidal carbon dioxide (EtCO_2_), WOB, and LTC were also extracted afterward from an ICU electronic medical record system, RPM-7400 (Nihon Koden, Tokyo, Japan). All parameters were measured each minute, and the average of the last 30 min of the observation period was recorded.

### Statistical analysis

We calculated that a minimum of eight patients in each group would be required to have 80% power to detect a difference in LTC of 30 mL/cmH_2_O between the success and failure groups at a significance level of 0.05. In the literature, the LTC in healthy adults is 80–100 mL/cmH_2_O [35], and in acute respiratory distress syndrome (ARDS) or cardiogenic pulmonary edema, the LTC is 29–42 mL/cmH_2_O [35–37]. The standard deviations of the LTC are reported to be 7–13 mL/cmH_2_O [36, 37]. Because LTC data for target patients were not available, we used a difference of 30 mL/cmH_2_O. Categorical data were analyzed using Fisher’s exact test. For the other parameters, a Mann-Whitney *U* test was used to compare the success and failure groups. Receiver operating characteristic (ROC) curve analysis was performed to evaluate the clinical implications of parameters. The area under the curve (AUC) was calculated to assess the discriminative power of the parameters. The analysis was performed using JMP Pro version 13.2.1 (SAS Institute Inc., Cary, NC, USA). This study was approved by the Ethical Review Board, Faculty of Medicine, Kagawa University (Heisei 29-049). The authors have no conflicts of interest to declare.

## Results

A total of 823 patients older than 18 years of age were admitted to the SICU during the target period. Among them, 503 patients were not mechanically ventilated during the SICU stay. According to the exclusion criteria, 142 patients were excluded. A total of 178 patients underwent the SBT process; however, 5 patients underwent a tracheotomy because they were judged to be unable to meet the extubation criteria by ICU members. Finally, we analyzed 173 patients and compared the success and failure groups. No patient had serious respiratory illness before the surgery or at ICU admission.

The subjects consisted of 65.9% men and 34.1% women, with a mean age of 68.2 years. Most (98.3%) were surgical patients, 90 (52.0%) were cardiac patients, 34 (19.7%) were craniocervical patients, and 16 (9.2%) were gastrointestinal surgery patients. Most patients (162, 93.6%) were extubated successfully, and 11 (6.4%) failed the extubation. In the failure group, three (27.3%) patients used NPPV, and eight (72.3%) were reintubated (Table 2). Four patients failed due to sputum clogging, three had pulmonary edema, two had hypercapnia, and one had aspiration pneumonia, and another was hypoxemic. The ventilation period and SBT number were 1651 ± 3011 min (mean ± SD) and 1 ± 1.7 (median ± SD) in the success group and 2330 ± 3797 min and 2 ± 2.9 in the failure group, respectively. There was no difference between the groups. Only vascular surgery and WOB were significantly associated with extubation failure (Table 2). The mean age and sex ratio was not different between the two groups. Among patient parameters, the APACHE II score, HR, RR, TV, RSBI, and LTC were significantly different. The average LTC values in the success and failure groups were 71.9 ± 23.0 and 43.3 ± 14.6, respectively, which were significantly different (*p* < 0.0001) (Table 3).

**Table 2:** Subject backgrounds

	Total	Success	Failure	*p* value
	*n* = 173	*n* = 162	*n* = 11	
Sex, *n* (%)				
Male	114 (65.9)	107 (66.1)	7 (63.6)	0.553
Female	59 (34.1)	55 (34.0)	4 (36.4)	0.696
Age (mean ± SD)	68.2 ± 12.8	68.2 ± 12.9	69.3 ± 11.7	0.854
Surgery, *n* (%)	170 (98.3)	160 (98.8)	10 (90.9)	0.179
Cardiac	90 (52.0)	86 (53.1)	4 (36.4)	0.519
Craniocervical	34 (19.7)	34 (21.0)	0 (0.0)	0.215
Gastrointestinal	16 (9.2)	13 (8.0)	3 (27.3)	0.064
Vascular	14 (8.1)	11 (6.8)	3 (27.3)	0.031
Other surgery	16 (9.2)	16 (9.9)	0 (0.0)	0.601
Others, *n* (%)	3 (1.7)	2 (1.2)	1 (9.1)	0.989
Emergency surgery, *n* (%)	14 (8.1)	13 (8.0)	1 (9.1)	0.616
Ventilation period, min (mean ± SD)	1694 ± 3058	1651 ± 3011	2330 ± 3797	0.516
SBT times (median ± SD)	1 ± 1.8	1 ± 1.7	2 ± 2.9	0.158
WOB*, J/L (mean ± SD)	0.77 ± 0.27	0.76 ± 0.26	0.99 ± 0.41	0.015
HFNC, *n* (%)	123 (71.1)	112 (69.1)	11 (100)	0.026
NPPV, *n* (%)	3 (1.7)	0 (0.0)	3 (27.3)	0.0002

The Mann-Whitney *U* test and Fischer’s exact test were applied. *WOB is a calculated estimate

*SBT* spontaneous breathing trial, *WOB* work of breathing, *HFNC* high-flow nasal cannula, *NPPV* non-invasive positive-pressure ventilation

**Table 3:** Comparison of parameters between the success group and failure group

	Total	Success	Failure	*p* value
	*n* = 173	*n* = 162	*n* = 11	
Female, *n* (%)	59 (34.1)	55 (34.0)	4 (36.4)	0.696*
Age (years)	68.2 ± 12.8	68.2 ± 12.9	69.3 ± 11.7	0.854
APACHE II score	17.6 ± 5.7	17.2 ± 5.3	23.4 ± 8.7	0.015
BMI	23.9 ± 4.2	24.1 ± 4.2	22.1 ± 3.8	0.075
HR (bpm)	77 ± 13.5	76.3 ± 13.1	87.1 ± 15.6	0.018
RR (/min)	16.1 ± 4.7	15.7 ± 4.1	23.3 ± 7.0	0.000
TV (mL)	457.4 ± 102.3	461 ± 98.8	405.4 ± 140.6	0.031
RSBI	38.4 ± 18.3	36.6 ± 15.7	64.3 ± 31.8	0.002
PEEP (cmH2O)	6.1 ± 1.4	6.2 ± 1.3	5.3 ± 2.4	0.065
SpO2 (%)	99.2 ± 1.0	99.2 ± 0.98	98.6 ± 1.4	0.076
EtCO2 (mmHg)	39.3 ± 5.2	39.4 ± 4.8	37.8 ± 9.3	0.828
LTC (mL/cmH2O)	70.1 ± 23.6	71.9 ± 23.0	43.3 ± 14.6	< 0.0001

Mann-Whitney *U* Test was applied. *Fischer’s exact test was applied for the comparison of ratios. From age to LTC, data were expressed as mean ± standard deviation

*APACHE II score* Acute Physiology and Chronic Health Evaluation II score, *BMI* body mass index, *HR* heart rate, *RR* respiratory rate, *TV* tidal volume, *RSBI* rapid shallow breathing index, *PEEP* positive end-expiratory pressure, *SpO2* arterial oxygen saturation, *EtCO2* end-tidal carbon dioxide, *LTC* lung-thorax compliance

**Fig. 3:**
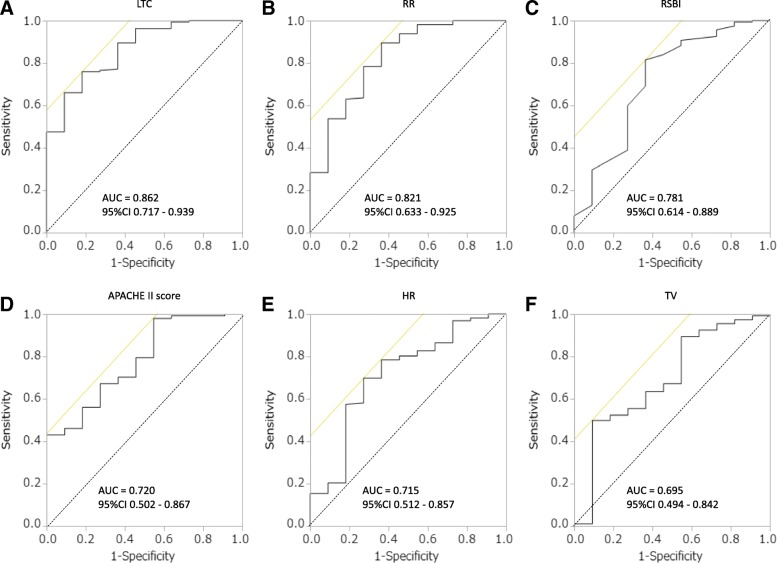
ROC curves for LTC (**a**), RR (**b**), RSBI (**c**), APACHE II score (**d**), HR (**e**), and TV (**f**) used to distinguish the success group from the failure group. In the ROC curve analysis, the AUC was highest for LTC (0.862), followed by the RR (0.821), RSBI (0.781), APACHE II score (0.720), HR (0.715), and TV (0.695). ROC curve: receiver operating characteristic curve; LTC: lung-thorax compliance; RR: respiratory rate; RSBI: rapid shallow breathing index; APACHE II score: Acute Physiology and Chronic Health Evaluation II score; TV: tidal volume; AUC: area under the curve, CI: confidence interval

In the ROC curve analysis, the AUC was highest for LTC (0.862), followed by the RR (0.821), RSBI (0.781), APACHE II score (0.720), HR (0.715), and TV (0.695) (Fig. 3).

## Discussion

LTC measured during the SBT was highly associated with extubation failure. The AUC of LTC was 0.862, which was highest among the parameters; therefore, LTC may be a good predictor of extubation failure. We compared the sensitivity and specificity of each parameter at the highest value of the Youden index [38], which was defined by the following formula: (sensitivity + specificity − 1). The maximum value of the Youden index was used as a criterion for selecting the optimum cutoff point of diagnostic tests [38]. At a cutoff point of 54, LTC had a moderate degree of sensitivity and a high degree of specificity. Considering patient safety, higher specificity is desirable, which indicates the usefulness of LTC as a predictor of extubation failure (Table 4). The sensitivity, specificity, and success rate of extubation were estimated at the different cutoff points for LTC. At a cutoff of 60, the estimated specificity was greater than 0.9, and the success rate was 96%. However, at a cutoff of 50, the estimated specificity was 0.636, and the success rate was 90%. LTC under 50–60 could indicate an increased probability of extubation failure in the SICU (Table 5).

**Table 4:** Sensitivity and specificity at the cutoff of the highest Youden index

	Cutoff	Sensitivity	Specificity
LTC (mL/cmH2O)	54	0.759	0.818
RR (/min)	21	0.895	0.636
RSBI	72	0.982	0.455
APACHE II	21	0.815	0.636
HR (bpm)	81	0.698	0.727
TV (mL)	451	0.500	0.909

Youden index = sensitivity + specificity − 1

*LTC* lung-thorax compliance, *RR* respiratory rate, *RSBI* rapid shallow breathing index, *APACHE II score* Acute Physiology and Chronic Health Evaluation II score, *HR* heart rate, *TV* tidal volume

**Table 5:** Estimates of sensitivity, specificity, and success rate at several LTC cutoffs

Cutoff	Sensitivity	Specificity	Success rate (%)
35	0.994	0.364	66
40	0.944	0.546	77
45	0.895	0.636	84
50	0.840	0.636	90
55	0.753	0.818	94
60	0.654	0.909	96
65	0.562	0.909	98
70	0.488	0.909	99

*LTC* lung-thorax compliance

Risk factors for extubation failure are known and include an RSBI greater than 100 [39–42], a PaO_2_ to FiO_2_ ratio less than 200 mmHg [40], PaCO_2_ greater than 44 mmHg during the SBT [43], and others [3, 11, 14, 15, 39–49]. In this study, vascular surgery was also a risk factor. In addition, WOB showed a significant difference between success group (0.76 ± 0.26) and failure group (0.99 ± 0.41). WOB is calculated using the following formula based on LTC: WOB = TV/LTC + (inspiratory flow velocity) × (resistance of the respiratory tract). The normal range of WOB is 0.3–0.7 J/L. The range is narrow, and it is more difficult to judge for determining extubation compared with LTC. In contract, LTC measured during the SBT is a candidate factor for extubation assessment because it is simple and accurate.

We adopted strict criteria in the SBT process because most of the subjects were operable patients and did not have severe respiratory complications. This may be the reason why the failure rate of our study (6.4%) was lower than those of previous reports (11–19%) [1–4, 10–15]. The APACHE II score, HR, RR, TV, and RSBI were significantly different between the two groups even though all subjects met the SBT process criteria. These parameters were recorded as the average of the last 30 min of the observation period. Furthermore, the sensitivities of the RR, RSBI, APACHE II score, and HR were 0.895, 0.982, 0.815, and 0.698, respectively, and the specificities were 0.636, 0.455, 0.636, and 0.727, respectively. The averages of the RR, RSBI, APACHE II score, or HR in the last 30 min of the observation period might be a predictor of extubation failure even when patients meet the SBT process criteria.

During the SBT process, five patients were excluded because they underwent a tracheostomy instead of extubation. The averages of the LTC, RR, RSBI, APACHE II score, and HR in these patients were 45.3, 22.9, 79.7, 18.8, and 75.4, respectively. All data indicated that patients in the failure group were in a worse condition than those in the success group. The results of the analysis were not different even with the inclusion of these patients.

According to Sandy et al., no difference was observed in the rate of extubation failure, duration of mechanical ventilation, or ICU and hospital stays among the SBT using PAV, T-tube, and PSV [50]. Bosma et al. revealed that the SBT using PAV was superior to PSV regarding the duration of mechanical ventilation or ICU stays [51]. Their results suggested that the PAV mode was a valid alternative for use in an SBT. Because PAV shows good synchronization with patients’ spontaneous breathing [23–25, 51], an SBT can be performed when the sedative drugs are reduced, which may allow more accurate measurement of LTC. Moreover, in the PAV mode, LTC can be measured continuously. As a problem of PAV, patients with interstitial pneumonia and ARDS are over-ventilated because the inspiratory flow is fast as a result of restrictive disorders, but the inspiratory time and TV are limited. In patients with severe chronic obstructive pulmonary disease (COPD) and ICU-acquired weakness (ICUAW) [52], respiratory muscle fatigue lowers the work of breathing and inspiratory flow. Ventilation becomes insufficient, and excess CO_2_ leads to hypercapnia. Conducting an SBT using the PAV mode in these patients with severe respiratory failure is difficult. However, short-term use of PAV is possible for LTC measurement. Further studies on the effectiveness of LTC measurement for patients with severe respiratory disease are necessary.

This study has several limitations. First, the study was conducted in a single center with a small sample size. Therefore, a generalization of the results may not be possible. Second, most of the subjects were surgical patients because the study was conducted in the SICU; few patients had serious respiratory diseases. However, the number of subjects in each group provided sufficient power for adequate statistical calculations, and the study was conducted in a single SICU, resulting in less heterogeneity in patient management and monitoring. Third, because the LTC data were indicated on the monitor of the ventilator, intensivists could view the data. However, the influence of this on our study was low because this study was designed as a retrospective cohort study.

## Conclusion

LTC measured during an SBT is a potential indicator of extubation failure in postoperative patients. Even in patients who met the strict SBT process criteria, the average RR, RSBI, APACHE II score, and HR in the last 30 min of the observation period might be the predictors of extubation failure. Further studies are necessary to determine the efficacy of this process for patients with severe respiratory disease.

## Abbreviations

APACHE II score: Acute Physiology and Chronic Health Evaluation II score
ARDS: Acute respiratory distress syndrome
AUC: Area under the curve
BMI: Body mass index
CNS: Central nervous system
COPD: Chronic obstructive pulmonary disease
EtCO_2_: End-tidal carbon dioxide
FiO_2_: Inspired oxygen fraction
HFNC: High-flow nasal cannula
HR: Heart rate
IV-PCA: Intravenous patient-controlled analgesia
LTC: Lung-thorax compliance
NPPV: Non-invasive positive-pressure ventilation
NSAIDs: Nonsteroidal anti-inflammatory drugs
PaCO_2_: Partial pressure of arterial carbon dioxide
PaO_2_: Partial pressure of arterial oxygen
PAV: Proportional assist ventilation
PEEP: Positive end-expiratory pressure
pH: Power of hydrogen
PNB: Peripheral nerve block
RASS: Richmond Agitation-Sedation Scale
ROC curve: Receiver operating characteristic curve
RR: Respiratory rate
RSBI: Rapid shallow breathing index
SBT: Spontaneous breathing trial
SICU: Surgical intensive care unit
SIMV: Synchronized intermittent mandatory ventilation
SpO_2_: Arterial oxygen saturation
TV: Tidal volume
WOB: Work of breathing
